# Supplementary material for: Niche partitioning facilitates coexistence of three apex predators in the Lapchi Valley, Central Himalaya, Nepal
Source: PLoS One. 2026 Apr 1;21(4):e0344947. doi: 10.1371/journal.pone.0344947 (PMC13042819; doi:10.1371/journal.pone.0344947)
Supplement: Attachment — Submitted filename: Response to editor and Reviewers.docx [file pone.0344947.s002.docx]

**Cover letter**

**Editor-in-Chief	Date:** January 5, 2026
PLOS ONE
Dear Editor,

Thank you for the opportunity to revise our manuscript entitled “Niche Partitioning Facilitates Coexistence of Three Apex Predators in the Lapchi Valley, Central Himalaya, Nepal.” We sincerely appreciate the editorial guidance and constructive comments provided by you and the reviewers.

In the revised manuscript, we have carefully addressed and incorporated all suggested corrections and feedback from the editor and reviewers. All comments have been responded to thoroughly, and the manuscript has been revised accordingly. We have provided detailed on amended funding statement, role of funders and, point-by-point responses to each comment to clearly indicate how the concerns were addressed.

We hope that the revisions meet the journal’s expectations and we appreciate your continued consideration of our work.

**Amended Funding Statement**

This study was supported by a faculty grant from the University Grants Commission (UGC), Nepal (FRG 77/78 S&T-01 2020/21 and FRG 080/81 S&T-07), the Andrew Sabin Family Foundation, Zoo New England, and the China Exploration and Research Society, Hong Kong. NPK’s effort was partially supported by an ANSO–CAS Visiting Scientist Fellowship at the Kunming Institute of Zoology, Chinese Academy of Sciences. RCK’s effort was supported in part by the Office of Research Infrastructure Programs (ORIP) of the National Institutes of Health through grant number P51OD010425 to the Washington National Primate Research Center, USA. There was no additional external funding received for this study.

**Role of the Funders**

The funders had no role in study design, data collection and analysis, decision to publish, or preparation of the manuscript.

We respectfully request that these amended statements replace the corresponding information in the online submission system, as suggested.

All authors have read and approved the revised submission. The manuscript is original, has not been published previously, and is not under consideration elsewhere. We declare no competing interests.

Thank you for your time and consideration. We look forward to your response.

Sincerely,

**Narayan Prasad Koju, PhD**
(On behalf of all co-authors)
Center for Postgraduate Studies, Nepal Engineering College, Pokhara University, Lalitpur, Nepal
Email: [npkoju.2003@gmail.com](mailto:npkoju.2003@gmail.com) / [narayanpk@nec.edu.np](mailto:narayanpk@nec.edu.np)

PONE-D-25-54236

**Comments from Editor**

When submitting your revision, we need you to address these additional requirements.

1. Please ensure that your manuscript meets PLOS ONE's style requirements, including those for file naming. The PLOS ONE style templates can be found at

<https://journals.plos.org/plosone/s/file?id=wjVg/PLOSOne_formatting_sample_main_body.pdf>

[https://journals.plos.org/plosone/s/file?id=ba62/PLOSOne_formatting_sample_title_authors_affiliations.pdf](https://track.editorialmanager.com/CL0/https:%2F%2Fjournals.plos.org%2Fplosone%2Fs%2Ffile%3Fid=ba62%2FPLOSOne_formatting_sample_title_authors_affiliations.pdf/1/010f019b28e3b31b-4be17d04-cfdb-4e60-8cf3-3b00f8c34520-000000/3I0imNICMBvg7VUNTIn_BEq3p1IExBtX_36o6ql1L6s=241).

Response: Thank you for support and consideration. We have carefully reviewed and revised the manuscript to ensure full compliance with PLOS ONE’s formatting and style requirements. The official PLOS ONE templates for the main text and for the title, authors, and affiliations were used. File naming conventions have also been corrected accordingly.

**Response: Thank you for the guidance. We have revised the manuscript to fully comply with PLOS ONE’s formatting and style requirements, including use of the official templates and correct file naming.2. Thank you for stating in your Funding Statement:**

“This study was supported by a faculty grant from the University Grants Commission (UGC), Nepal (FRG 77/78 S&T-01 2020/21 and FRG 080/81 S&T-07), Andrew Sabin Family Foundation, Zoo New England, and the China Exploration and Research Society, Hong Kong. RCK’s effort was supported in part by the Office of Research Infrastructure Programs (ORIP) of the National Institutes of Health through grant number P51OD010425 to the Washington National Primate Research Center, USA”

Please provide an amended statement that declares *all* the funding or sources of support (whether external or internal to your organization) received during this study, as detailed online in our guide for authors at <http://journals.plos.org/plosone/s/submit-now>.  Please also include the statement “There was no additional external funding received for this study.” in your updated Funding Statement.

Please include your amended Funding Statement within your cover letter. We will change the online submission form on your behalf.

**Response: We thank the Editor for this comment. The Funding Statement has been amended to clearly declare all sources of financial support received during the study following the feedback.**

3. Thank you for stating the following financial disclosure:

“This study was supported by a faculty grant from the University Grants Commission (UGC), Nepal (FRG 77/78 S&T-01 2020/21 and FRG 080/81 S&T-07), Andrew Sabin Family Foundation, Zoo New England, and the China Exploration and Research Society, Hong Kong. RCK’s effort was supported in part by the Office of Research Infrastructure Programs (ORIP) of the National Institutes of Health through grant number P51OD010425 to the Washington National Primate Research Center, USA”

Please state what role the funders took in the study.  If the funders had no role, please state: "The funders had no role in study design, data collection and analysis, decision to publish, or preparation of the manuscript."

If this statement is not correct you must amend it as needed.

Please include this amended Role of Funder statement in your cover letter; we will change the online submission form on your behalf.

**Response: The Funding Statement has been amended and statement** "The funders had no role in study design, data collection and analysis, decision to publish, or preparation of the manuscript" is added.

4. We note that your Data Availability Statement is currently as follows: [All relevant data are within the manuscript and its Supporting Information files]

Please confirm at this time whether or not your submission contains all raw data required to replicate the results of your study. Authors must share the “minimal data set” for their submission. PLOS defines the minimal data set to consist of the data required to replicate all study findings reported in the article, as well as related metadata and methods (<https://journals.plos.org/plosone/s/data-availability#loc-minimal-data-set-definition>).

For example, authors should submit the following data:

- The values behind the means, standard deviations and other measures reported;

- The values used to build graphs;

- The points extracted from images for analysis.

Authors do not need to submit their entire data set if only a portion of the data was used in the reported study.

If your submission does not contain these data, please either upload them as Supporting Information files or deposit them to a stable, public repository and provide us with the relevant URLs, DOIs, or accession numbers. For a list of recommended repositories, please see <https://journals.plos.org/plosone/s/recommended-repositories>.

If there are ethical or legal restrictions on sharing a de-identified data set, please explain them in detail (e.g., data contain potentially sensitive information, data are owned by a third-party organization, etc.) and who has imposed them (e.g., an ethics committee). Please also provide contact information for a data access committee, ethics committee, or other institutional body to which data requests may be sent. If data are owned by a third party, please indicate how others may request data access.

**Response: We confirm that our submission contains the minimal data set required to replicate all results reported in the study. All supplementary filed, associated metadata and R code have been deposited in Zenodo. The data and code are publicly available at:** **<https://doi.org/10.5281/zenodo.18053588> . The Data Availability Statement has been updated accordingly.**

5. We note that Figures 1 & 2 in your submission contain [map/satellite] images which may be copyrighted. All PLOS content is published under the Creative Commons Attribution License (CC BY 4.0), which means that the manuscript, images, and Supporting Information files will be freely available online, and any third party is permitted to access, download, copy, distribute, and use these materials in any way, even commercially, with proper attribution. For these reasons, we cannot publish previously copyrighted maps or satellite images created using proprietary data, such as Google software (Google Maps, Street View, and Earth). For more information, see our copyright guidelines: <http://journals.plos.org/plosone/s/licenses-and-copyright>.

We require you to either (1) present written permission from the copyright holder to publish these figures specifically under the CC BY 4.0 license, or (2) remove the figures from your submission:

a. You may seek permission from the original copyright holder of Figures 1 &2to publish the content specifically under the CC BY 4.0 license.

We recommend that you contact the original copyright holder with the Content Permission Form (<http://journals.plos.org/plosone/s/file?id=7c09/content-permission-form.pdf>) and the following text:

“I request permission for the open-access journal PLOS ONE to publish XXX under the Creative Commons Attribution License (CCAL) CC BY 4.0 (<http://creativecommons.org/licenses/by/4.0/>). Please be aware that this license allows unrestricted use and distribution, even commercially, by third parties. Please reply and provide explicit written permission to publish XXX under a CC BY license and complete the attached form.”

Please upload the completed Content Permission Form or other proof of granted permissions as an "Other" file with your submission.

In the figure caption of the copyrighted figure, please include the following text: “Reprinted from [ref] under a CC BY license, with permission from [name of publisher], original copyright [original copyright year].”

b. If you are unable to obtain permission from the original copyright holder to publish these figures under the CC BY 4.0 license or if the copyright holder’s requirements are incompatible with the CC BY 4.0 license, please either i) remove the figure or ii) supply a replacement figure that complies with the CC BY 4.0 license. Please check copyright information on all replacement figures and update the figure caption with source information. If applicable, please specify in the figure caption text when a figure is similar but not identical to the original image and is therefore for illustrative purposes only.

The following resources for replacing copyrighted map figures may be helpful:

USGS National Map Viewer (public domain): <http://viewer.nationalmap.gov/viewer/>

The Gateway to Astronaut Photography of Earth (public domain): <http://eol.jsc.nasa.gov/sseop/clickmap/>

Maps at the CIA (public domain): <https://www.cia.gov/library/publications/the-world-factbook/index.html> and <https://www.cia.gov/library/publications/cia-maps-publications/index.html>

NASA Earth Observatory (public domain): <http://earthobservatory.nasa.gov/>

Landsat: <http://landsat.visibleearth.nasa.gov/>

USGS EROS (Earth Resources Observatory and Science (EROS) Center) (public domain): http:

 Earth (public domain): [http://www.naturalearthdata.com/](http://track.editorialmanager.com/CL0/http:%2F%2Fwww.naturalearthdata.com%2F/1/010f019b28e3b31b-4be17d04-cfdb-4e60-8cf3-3b00f8c34520-000000/PA5-CCt4A_ibcKuhof-4Xxquh-UxldH1mwO6UUlWOOI=241)

**Response: Thank you for raising this copyright concern. The Figure 1 was generated by the authors using the ESRI Global Land Cover dataset obtained from the ESRI Living Atlas (**[**https://livingatlas.arcgis.com/landcover/**](https://livingatlas.arcgis.com/landcover/)**). This dataset is distributed under the Creative Commons Attribution (CC BY 4.0) license, and no proprietary sources (e.g., Google Maps/Earth) were used. The figure caption has been updated to include proper attribution.**

**Figure 2 was created using a Digital Elevation Model (DEM) downloaded from USGS (**[**http://viewer.nationalmap.gov/viewer/**](http://viewer.nationalmap.gov/viewer/)**), which is publicly available and compatible with the CC BY 4.0 license**. **In addition, Figure 2 has been revised in the revised manuscript following Reviewer 2’s comments. Accordingly, both figures comply with PLOS ONE’s CC BY 4.0 licensing requirements, therefore no additional permissions are required.**

6. Please remove your figures from within your manuscript file, leaving only the individual TIFF/EPS image files, uploaded separately. These will be automatically included in the reviewers’ PDF.

**Response: All figures are submitted in TIFF and removed from manuscript file as per comment in revised submission.**

7. If the reviewer comments include a recommendation to cite specific previously published works, please review and evaluate these publications to determine whether they are relevant and should be cited. There is no requirement to cite these works unless the editor has indicated otherwise.

**Response: There is no specific citation recommended by the reviewers.**

Reviewers' comments:
Please use the space provided to explain your answers to the questions above. You may also include additional comments for the author, including concerns about dual publication, research ethics, or publication ethics. (Please upload your review as an attachment if it exceeds 20,000 characters)

**Reviewer #1:**

General comment: The manuscript addresses an important question about the coexistence of large carnivores. However, the strength of the inferences is limited by (i) the lack of explicit hypotheses/predictions, (ii) inconsistencies and omissions in the design/sampling, and (iii) spatial/temporal conclusions based on very simple methodological decisions without sensitivity analysis. I recommend major revision.
1) Introduction
Hypotheses and predictions are missing. It remains to be clarified what patterns are expected (spatial, temporal, and trophic) and what mechanisms sustain them (competition, exploitation vs. interference, anthropogenic subsidies, prey availability).
I recommend adding hypotheses and predictions

**Response: We thank the reviewer for this constructive and insightful comment. We agree that explicitly stating hypotheses and predictions is essential to strengthen inference and clarify the analytical framework. In response, we have substantially revised the Introduction to frame the study as hypothesis-driven and to explicitly link our analyses to ecological mechanisms underlying carnivore coexistence. Precisely, we added a new paragraph at the end of the Introduction that clearly pronounces hypotheses and predictions related to spatial, temporal, and trophic niche partitioning among snow leopards, leopards, and Himalayan wolves.**

.2) Methodology – Design and effort
-Inconsistencies in number of sites/cameras/duration. Twenty-six cameras in 20 locations are mentioned, but Figure 1 shows 19 locations. In addition, the operating time is reported first, followed by the number of cameras (reverse order).
I recommend: (a) standardizing figures in text/figures/tables; (b) describing whether there was >1 camera per location and under what criteria; (c) presenting an effort table with camera days per location and per year, failures, and relocations. The experimental design should be better explained.
-Long study period (2018–2025) without stratification. They report an average of ~761 camera days, which implies that cameras were not active throughout the entire period and possible temporal bias in location.
I recommend: (a) detailing the actual windows of activity for each camera; (b) indicating whether the spatial distribution changed between years; (c) stratifying analyses (or including covariates) by year/season to avoid mixing ecologically dissimilar periods that inflate overlaps.
-Camera model and recording mode. The exact model is requested (line 98). Further on (line 166), it is indicated that there were images and videos, but earlier only images are mentioned.
I recommend specifying the brand/model, sensitivity/intervals, and for each period whether photos, videos, or both were captured, and how they were integrated into the analysis.

**Response: We thank the reviewer for highlighting these important points. In response:**

1. **Camera trap locations: The missing camera trap has been added in the revised Figure 1, ensuring consistency between the text and figure.**
2. **Camera trap effort and design: We have added a supplementary table detailing camera trap installation dates, active periods, removal dates, and total working hours for each camera. This table provides camera days per location and per year, including information on camera failures and relocations, clarifying the experimental design and effort across the study period.**
3. **Camera model and settings: We used Bushnell Trophy Cam HD Trail Cameras (Essential E3 16MP and E2 12MP). Cameras were set to hybrid mode (three photos followed by a 10-second video) with a trigger interval of 1 second and sensitivity set to low. These settings were applied consistently across the study period, and both images and videos were integrated into the analysis.**

3) Methodology – Space and time
-Spatial overlap is calculated using 100 m buffers around detections and altitude limits defined with the same data. This approach is overly simplistic and potentially circular.
I recommend: (a) replacing or supplementing with use estimators (e.g., use density/kernel)); (b) reporting sensitivity analyses to buffer width and altitude limits

**Response: We are thankful for both reviewers for the suggestion. Following the reviewers’ (including reviewer #2) suggestions, we applied Minimum Convex Polygon (MCP) analysis to more robustly quantify the spatial distribution and overlap of the three predators. During MCP construction, we accounted for elevation and land cover, including high steep mountain areas, to ensure realistic habitat representation. This approach supplements our previous presence-based method, reducing potential circularity from using the same data to define buffers and altitude limits. We have updated the Results and Discussion sections to reflect these revised analyses.**

-Temporal activity collapsed into 7 years. It is not appropriate to speak of “synchrony” of the three predators with multi-year averaged data.
Request: (a) report Δ with CI and n per pair; (b) stratify by year/season or add a supplementary analysis showing the temporal stability of the patterns.

**Response: We thank the reviewer for highlighting this important point. In response, we have reported Δ values with confidence intervals (CI) and sample sizes (n) for each predator pair, which are now presented in Table 1 and Table 2. Additionally, to address concerns regarding temporal averaging, we have stratified the analyses by season and included a supplementary analysis showing the seasonal activity patterns of the three-predator species. Its detail is submitted in Supplementary figure 2 and Supplementary table 2.**

4) Methodology – Diet
-Sample sizes by species not reported. To interpret trophic overlap, the number of feces analyzed per species (DNA/micro-histology) and the criteria for selecting the histological “subset” are needed.

**Response: We thank the reviewer for this observation. Information regarding sample sizes and species identification is provided in the Predator Identification section of the manuscript (last paragraph). “*Of the 93 DNA extracts analyzed, 14 were confirmed as snow leopard, 11 as leopard, and 18 as Himalayan wolf. Additionally, 18 samples were identified as red fox, 13 as leopard cat, while 19 samples failed to amplify with the primers”*.**

5) Results
-Temporal synchrony (l. 260–261): Avoid statements of synchrony for the three predators without temporal stratification.
-Spatial overlap: Strong conclusions are based on buffer + altitudinal clipping.
-Diet: Include n per species analyzed and Pianka's CI (bootstrap per scat).
To support the Discussion and conclusions, the manuscript should: (i) state hypotheses/predictions and mechanisms; (ii) clarify and tabulate design/effort (with temporal stratification); (iii) replace or, at least, subject to sensitivity analysis the spatial analysis based on buffers; and (iv) strengthen the diet section with n per species. These improvements are necessary to ensure reproducibility and inferential validity.

**Response:** **We thank the reviewer for these constructive comments, which have helped us strengthen the rigor and clarity of the manuscript. In response to concerns regarding spatial overlap, we have revised the analysis and generated a new spatial overlap map using Minimum Convex Polygon (MCP) methods, replacing the buffer-based approach. This revision follows the recommendations of Reviewer #2; the corresponding methods, results, and figures have been updated accordingly. In the diet analysis, we have clearly reported the sample size (n) per predator species. In addition, we have added the inference on dietary overlap by calculating Pianka’s niche overlap index with associated 95% confidence intervals. More broadly, to better support the discussion and conclusions, we have clarified the study’s hypotheses, predictions, and underlying ecological mechanisms; tabulated sampling design and effort with explicit temporal stratification; and revised the spatial and dietary analyses to improve reproducibility and inferential validity. These changes are reflected throughout the revised manuscript.**

**Reviewer #2: Comments to authors**

I read with great interest your article titled “Niche Partitioning Facilitates Coexistence of Three Apex Predators in the Lapchi Valley, Central Himalaya, Nepal.” This study examines niche partitioning and coexistence among sympatric snow leopards, leopards, and Himalayan wolves in the Lapchi Valley of Central Nepal. Using camera trapping and dietary analyses, the authors assess spatial, temporal, and trophic overlap to explore mechanisms facilitating predator coexistence in a high-altitude Himalayan system. My primary concerns are related to some of the methods and the depth of the introduction and discussion given the nature of the topic and approaches.
Please see my specific line comments below.
Abstract
In the abstract, it would be helpful to start with one or two sentences that provide some context and clearly point out the main gap before moving into the study objectives.

**Response: Few sentences “Large carnivores increasingly coexist in human-modified mountain landscapes, yet empirical evidence on how multiple apex predators partition space, time, and resources to reduce competition remains limited, particularly in the Central Himalaya. Understanding these mechanisms is critical for predicting interspecific interactions and informing conservation in resource-limited alpine ecosystems.” is added in abstract following feedback**

Introduction
The introduction currently reads thin and would benefit from further development. Lines 47–54 discuss the threats these apex predators face, but the content is split across two paragraphs and would benefit from better organization.
While some of these points were briefly mentioned, the authors should further expand on contexts such as the role of apex predators in the ecosystem, existing mechanisms of coexistence among apex predators (e.g., different types of niche partitioning), the remaining knowledge gaps, and what makes the study system unique, etc. Expanding on these areas would provide a stronger theoretical background for the study.

**Response: The section of the introduction is revised following the feedback.**

Methods
Please ensure consistent citation formatting.
The spatial overlap analysis adopts a presence-based, rule-constrained approach, conceptually similar to an environmental envelope method, rather than more complex approaches such as MCP, SDM, or JSDM. The authors should more explicitly justify their methodological choice and discuss its underlying assumptions, rather than adopting alternative approaches. Please ensure that index abbreviations are used consistently across the manuscript (e.g., “B” is used twice for different indices).

**Response: We thank the reviewer for this insightful comment regarding the spatial overlap analysis and its underlying assumptions. In response, we have revised the spatial analysis by adopting a Minimum Convex Polygon (MCP) approach to estimate species ranges and quantify spatial overlap. The revised MCP-based analysis is now fully described in the Methods, and the resulting overlap estimates and spatial representations are presented in the Results and associated figures. The Discussion has also been updated to reflect the assumptions and limitations of the MCP approach and to justify its suitability given our sample size and study objectives. The index abbreviation “B” is revised to “β”.**

Results
The spatial overlap results are derived from a relatively small number of camera trap sites (7–11 detections per species). Given this limited spatial sampling, stronger justification is needed when interpreting these estimates, particularly statements suggesting complete spatial overlap or subsumed distributions. The authors should clarify that these results reflect detection-based, potential overlap, and discuss how sampling design and buffer choices may influence the observed patterns, or alternatively consider using additional data or complementary methods to support their conclusions.

**Response: The results section has been revised in accordance with the reviewer’s feedback. Furthermore, this section now includes results derived from the Minimum Convex Polygon (MCP) analysis, which provide a clearer and more robust assessment of spatial overlap.**

Please ensure consistent table formatting. Please also correct the grammar in lines 299–301; there is an extra closing parenthesis.

**Response: Corrected**

Discussion
Please ensure consistent citation formatting throughout the manuscript.
The Discussion should be expanded and developed in greater depth, particularly with respect to the conservation implications of the findings. Given that both the topic and the analytical approaches are not particularly novel, and that similar analyses of apex and meso-predator interactions have been conducted across other regions of the Himalayas, it is especially important for the authors to clearly articulate how this study system differs from previous work and what new insights it provides. In particular, the authors should better explain why understanding niche partitioning among snow leopards, leopards, and Himalayan wolves in this specific area matters. For example, do the observed dietary patterns reflect differential coexistence strategies, human-predator relationships, or local perceptions of predators by herders? What about the spatial overlap results, and how do those ecological findings link to the cultural context? What are the potential long-term implications for prey resources, habitat use, or conflict risk given the high spatial and temporal overlap observed among these species?

**Response: All citations have been cross-checked and standardized following PLOS ONE guidelines.**

**The Discussion has been expanded to better highlight the conservation relevance of the findings and to clarify how this study adds insight beyond previous Himalayan research. We now emphasize the ecological and management significance of niche partitioning among snow leopards, leopards, and Himalayan wolves in the Lapchi Valley, particularly in relation to prey use, spatial overlap, and potential conflict risk. Although local perceptions of predators and herder attitudes were not directly assessed, this limitation is now clearly acknowledged, and the Discussion outlines how socio-cultural factors may interact with the observed ecological patterns.**
